# Penetration-enhancement underlies synergy of plant essential oil terpenoids as insecticides in the cabbage looper, *Trichoplusia ni*

**DOI:** 10.1038/srep42432

**Published:** 2017-02-09

**Authors:** Jun-Hyung Tak, Murray B. Isman

**Affiliations:** 1 Faculty of Land and Food Systems, University of British Columbia, Vancouver, British Columbia, V6T 1Z4, Canada

## Abstract

Many plant essential oils and their terpenoid constituents possess bioactivities including insecticidal activity, and they sometimes act synergistically when mixed. Although several hypotheses for this have been proposed, the underlying mechanism has not been fully elucidated thus far. In the present study, we report that in larvae of the cabbage looper, *Trichoplusia ni*, most synergistic or antagonistic insecticidal activities among mixtures of plant essential oil constituents are pharmacokinetic effects, owing to changes in solubility as well as spreadability on a wax layer. Among the major constituents of rosemary (*Rosmarinus officinalis*) oil, *in vitro* analysis revealed up to a 19-fold increase in penetration of camphor in a binary mixture with 1,8-cineole through the larval integument, suggesting increased penetration as the major mechanism for synergy. A total of 138 synergistic or antagonistic interactions among 39 compounds were identified in binary mixtures *via* topical application, and these were highly correlated to changes in surface tension as measured by contact angle of the mixtures on a beeswax layer. Among compounds tested, *trans*-anethole alone showed evidence of internal synergy, whereas most of remaining synergistic or antagonistic combinations among the three most active compounds were identified as penetration-related interactions, confirmed *via* a divided-application bioassay.

For over three decades, interest in botanical insecticides has been growing continuously. Within this area, scientific literature on plant essential oils has expanded rapidly over the past 15 years, becoming one of the mainstreams in botanical insecticide research^1^. Typically, plant essential oils are complex mixtures of mono- and sesquiterpenoids with as many as 100 constituents. This complexity often results in superior bioactivity of the ‘full mixture’ of the major constituents or the natural (crude) oils themselves compared to the isolated major compounds, including antibacterial activity^2^,^3^, enzyme inhibitory activity^4^, insect feeding deterrence^5^, repellence^6^, and acute insecticidal and miticidal activity^7^,^8^. Overall bioactivity of certain essential oils was restored by blending some of the major constituents (sometimes requiring minor constituents as well), indicating synergistic interactions among those constituents. Moreover, there are ample studies of synergistic combinations of other plant-derived constituents already reported^9^,^10^,^11^, suggesting the importance of understanding their mechanisms of synergy.

Owing to the structural and compositional chemical diversity of plant essential oil constituents, bioactivity has been attributed to multiple modes-of-action and/or sites-of-action. For example, potential antibacterial modes-of-action of plant essential oils include degradation of cell walls, damage to cytoplasmic membranes or membrane proteins, and coagulation or leakage of cell components^2^. As for insecticidal activity, receptors in the insect nervous system for neurotransmitters such as γ-aminobutyric acid (GABA), octopamine, tyramine, or acetylcholinesterase (AChE) are considered potential targets^12^,^13^. Moreover, since the discovery of synergistic activity of sesame oil to natural and synthetic insecticides by Eaglenson in 1940^14^, many essential oils and their constituents have been reported to inhibit activity of detoxifying enzymes, which can lead to synergistic toxicity with synthetic insecticides^15^,^16^,^17^. These two characteristics of plant essential oils have led to speculation that the synergistic toxicity of plant essential oils is a consequence of biochemical mechanisms, either *via* increased activation at the putative target site(s) or decreased metabolic detoxification. Some attempts have been made to prove these hypotheses, but most studies either failed to provide evidence or only partially explained the enhanced toxicity observed^18^,^19^,^20^.

We previously suggested a penetration-enhancing effect through the insect cuticle as a mechanism of synergy between 1,8-cineole and camphor, the two major constituents of rosemary (*Rosmarinus officinalis*) oil, and *in vivo* analysis showed increased internal concentrations of the synergistic combination^21^. However, since we reported this physicochemical aspect based on a single mixing ratio alone, and inhibition in detoxicative enzyme activity might also contribute to increased internal concentrations of the compounds, a detailed study was required to verify this effect, enhanced by an *in vitro* investigation focusing solely on the penetration of the compounds. Moreover, as there is a chance that this phenomenon is unique to rosemary oil, we expanded the pool of compounds for combination to test whether this might be a more general mechanism of synergy.

In the present study, the cabbage looper, *Trichoplusia ni* (Lepidoptera: Noctuidae), was selected as a robust model insect. It is a major agricultural pest widespread in the Americas, East Africa and some Asian countries attacking not only cruciferous crops such as cabbage, broccoli, and cauliflower, but also can be found on beets, celery, lettuce, and tomatoes^22^. Since the larvae cause direct damage to crops, they are the main target stage for pest management. We examined [1] *in vitro* penetration of the synergistic combinations of major constituents of rosemary oil, [2] insecticidal activity of 39 compounds generally found in plant essential oils as well as potential structure-activity relationships, [3] synergistic interactions among selected compounds and their mechanism of synergy, and [4] diagnostic methods to determine the involvement of penetration in synergistic combinations.

## Results

### Synergistic toxicity of 1,8-cineole and camphor in combination and their penetration through the insect integument

Ratio-dependent response in toxicity between the two major constituents of rosemary oil, 1,8-cineole and camphor, was examined in 3rd instar larvae of the cabbage looper (Table 1). Based on LD_50_ values, enhanced toxicity (compared to expected LD_50_ values) was observed only in the middle range of mixtures (1,8-cineole: camphor = 75:25 to 40:60), and the most toxic ratio was 60:40 (LD_50_ = 186.9 μg/insect). Interestingly, the divided application of the compounds eliminated any increased toxicity, showing that the toxicity can only be enhanced when the compounds are applied as a mixture.

**Figure 1:**
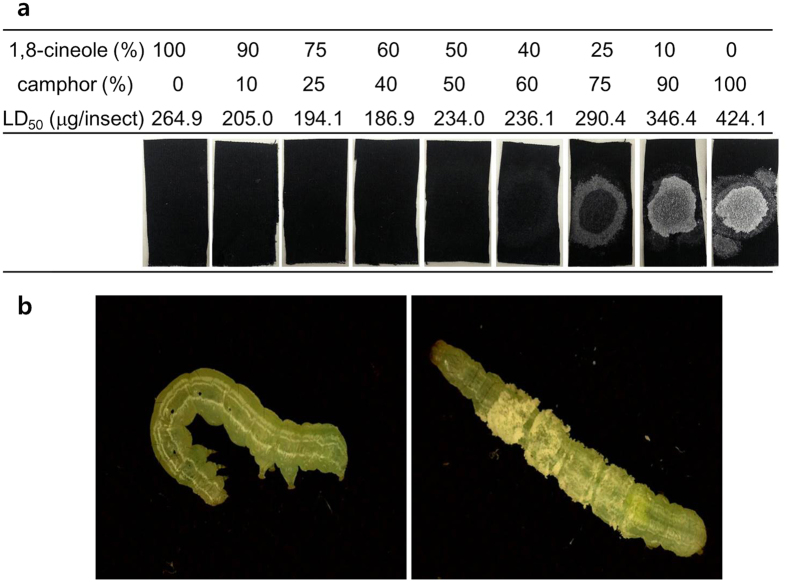
Direct correlation between insecticidal activity and solubility of camphor in binary mixtures with 1,8-cineole. (**a**) Application status of the binary mixture in different blending ratios, and (**b**) difference in recrystallization of camphor (left; 1,8-cineole:camphor = 75:25, right; camphor alone).

The increased toxicity of the binary mixture appears to be directly associated to the degree of penetration through the insect’s integument as a result of altered solubility of camphor. Camphor showed recrystallization as a visible white residue on black cotton fabric when its proportion in the mixture was increased, indicating decreased solubility. Moreover, the limited solubilization was also observed in the insect bioassay: while the binary mixture (1,8-cineole: camphor = 75:25) left no residue on the surface of larvae, topical application of camphor alone resulted in distinctive recrystallization on the insects’ cuticle (Fig. 1).

**Figure 2:**
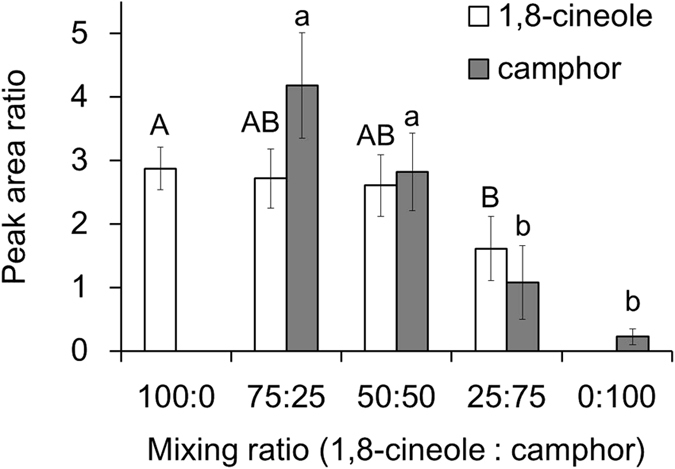
*In vitro* GC-MS analyses of the cuticular penetrations of 1,8-cineole and camphor in selected blending ratios.

Our *in vitro* analysis of penetration through the integument of 5th instar larvae confirmed that the more toxic combination had a relatively higher amount of camphor in the receiver solution (Fig. 2). As the concentration of camphor became greater in the binary mixture, penetration through the cuticle decreased, as did toxicity of the mixture. For example, the peak area ratio of individual camphor to the internal standard was 0.22 ± 0.12, whereas when the concentration of camphor was only one quarter (1,8-cineole: camphor = 75:25), the peak area ratio of camphor significantly increased to 1.05 ± 0.21 (relative area ratio of 4.18 ± 0.83, assuming the same amount was applied), indicating 4.7 times greater penetration through the integument.

### Insecticidal activity of 39 test compounds

Among the 39 tested compounds, thymol (25.7 μg/insect) was the most toxic compound based on LD_50_ values *via* topical administration to 3rd instar larvae of the cabbage looper, followed by carvacrol and α-terpineol (36.3 and 67.9 μg/insect, respectively, Table 2). Those three compounds also showed high internal toxicity when injected into the hemocoel of 5th instar larvae at 500 μg/insect (>80% mortality). Besides those three highly active compounds, there were several other potentially toxic compounds, including terpinen-4-ol, *trans*-cinnamaldehyde, eugenol, camphor and borneol. Although the latter were either mildly or weakly toxic *via* topical administration, their internal toxicity was comparable to the first three compounds, showing >75% mortality *via* injection. Among the latter compounds, camphor and borneol showed the greatest difference between external and internal toxicity. LD_50_ values for the topical administration of camphor and borneol were 602.6 and 759.7 μg/insect, whereas injection (500 μg/insect) resulted in 77 ± 7% and 80 ± 6% mortality, respectively.

Regarding structure-activity relationships, the contact angles on a beeswax layer as well as four other traditional parameters – octanol-water partition coefficient, surface tension, solvent accessible surface area and polar surface area – are shown in Table 2 and Supplementary Table S1. No notable correspondence to either topical toxicity or injection mortality of the compounds was found (R^2^ in linear regression <0.2).

### Combination effects of binary mixtures and their mechanism of synergy

Three of the most toxic compounds *via* topical administration, thymol, carvacrol and α-terpineol (group A), and another three which showed notable internal toxicity, eugenol, *trans*-cinnamaldehyde, and camphor (group B) were subjected to further study of their interactions with other compounds. Table 3 shows the list of the compounds that exhibited the greatest synergistic or antagonistic effects as well as the number of compounds that showed positive or negative interactions. Whereas only 6 compounds showed synergistic interactions with thymol in binary mixtures, three quarters of the tested compounds were able to increase the toxicity of camphor (30 out of 38 compounds). In terms of antagonistic interactions, thymol and *trans*-cinnamaldehyde had 11 antagonistic interactions with four overlapping compounds including borneol, citral, linalool oxide and verbenone, whereas none of the compounds significantly decreased the topical toxicity of camphor.

Similar to the combinations of 1,8-cineole and camphor, most of the synergistic interactions (exceptions: *trans*-anethole to thymol, *trans*-anethole and α-terpineol to carvacrol, terpinen-4-ol and methyl salicylate to α-terpineol) as well as all of the antagonistic interactions among group A compounds disappeared in the divided application (Supplementary Tables S2 to S4). This suggests that most of the synergistic or antagonistic interactions among those compounds are produced by increased or decreased penetration of the toxicants. Compared to group A, the synergistic interactions among group B compounds were more noteworthy. Not only was the number of synergistic interactions greater in group B, but also the degree of enhancement was superior compared to that for the compounds in group A. For example, the average χ^2^ values for the synergistic combinations of thymol, carvacrol and α-terpineol were 11.6, 17.9 and 40.3, respectively, whereas those for eugenol, *trans*-cinnamaldehyde and camphor were 48.8, 60.3 and 55.8, respectively. Moreover, the χ^2^ values from all the synergistic combinations including thymol and half of those with carvacrol were less than 20, but only 20% of the synergistic combinations in group B were less than 20 (16 out of 78 combinations).

Among the functional groups of synergizing compounds, hydrocarbons tend to provide the most significant boosting effect. For example, excepting *trans*-anethole, all of the boosting agents for thymol were hydrocarbons, including 3-carnene, *p*-cymene, limonene, α-terpinene and γ-terpinene (Supplementary Table S2). Also, all 6 compounds which showed complete mortality (100%) when mixed with eugenol were hydrocarbons. Further, all 8 hydrocarbons tested in the present study showed synergistic effects with all the group B compounds except camphene to camphor (see Supplementary Tables S5 to S7).

**Figure 3:**
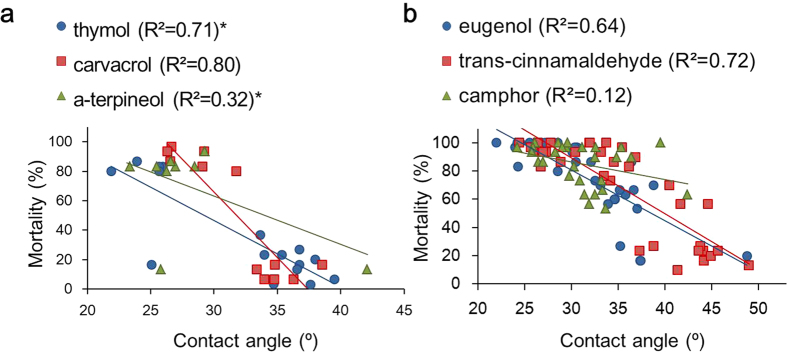
Interactions between mortality in the topical application and contact angles on a beeswax layer of selected mixtures. (**a**) The synergistic and antagonistic binary mixtures of group A compounds (thymol, carvacrol and α-terpineol) at LD_50_ and an equivalent amount of blending compounds, (**b**) the binary mixtures of group B compounds (eugenol, *trans*-cinnamaldehyde and camphor) at LD_20_ + LD_20_ with other compounds. *When camphene was excluded, the R^2^ values of thymol and α-terpineol increased to 0.91 and 0.83, respectively.

To understand the general principles as to how the physical properties of the compounds can contribute to the change in their toxicity, the four physical parameters of the mixtures as well as their contact angles on a beeswax layer were examined. Among the parameters, a modest correlation between the proportional average of polar surface area of the mixtures and their mortality was observed for thymol and carvacrol (R^2^ values of 0.62 and 0.64, respectively). In all other combinations, none of the parameters were correlated to any of the compounds in either group (R^2^ < 0.5). On the other hand, as shown in Fig. 3, contact angles of the mixtures on a beeswax layer were more strongly correlated with mortality in the topical assay. Most of the synergistic combinations showed lesser contact angles than those of the individual compounds, whereas the antagonistic ones had either greater or unchanged contact angles. In group A, camphene was the only compound that did not follow this general trend; although contact angles were decreased, its’ binary mixtures with thymol or α-terpineol produced antagonistic responses. The R^2^ values for thymol and α-terpineol were 0.71 and 0.32 respectively, but when camphene was excluded, these values increased to 0.91 and 0.83, respectively, indicating a strong correlation between contact angle and toxicity. In group B, eugenol and *trans*-cinnamaldehyde (R^2^ = 0.64 and 0.72) showed the same tendency of increased toxicity with lowered angles, and decreased toxicity with higher angles. Among the six sets of combinations tested in the present study, only camphor did not show a strong correlation to the contact angle change, with R^2^ value of 0.12. However, the general trend we identified still applied as in no case did a lowered contact angle result in decreased toxicity.

### Synergistic and antagonistic effects of the selected compounds to thymol and their penetration through the insect integument

Among the binary mixtures with thymol, *trans*-anethole showed a distinctive synergy, exhibiting increased toxicity not only in the mixture but also *via* divided application. A further study was conducted to understand the insecticidal activity in association with other selected compounds and their relationship to penetration through the integument. Based on the topical LD_50_ values of the binary mixtures, *trans*-anethole, 3-carene and α-terpinene showed strong synergistic interactions with thymol. Contact angles on a beeswax layer showed significant decreases with 3-carene and α-terpinene, but not with *trans*-anethole, suggesting its unique synergy (Table 4). Statistically, geranyl acetate and anisaldehyde exhibited synergistic and additive interactions with thymol, but they increased the actual amount of thymol in the LD_50_ values significantly compared to that of individual thymol, indicating antagonistic interactions. Both compounds significantly increased contact angles, suggesting reduced cuticular penetration of the toxicant, thymol.

Although not significantly different, both of the tertiary mixtures of thymol + *trans*-anethole + 3-carene or α-terpinene had greater reductions of thymol in the LD_50_ values (45.8% reduction on average, compared to individual thymol) compared to the binary mixtures or the tertiary mixture of thymol + 3-carene + α-terpinene. In the tertiary mixtures of thymol + *trans*-anethole and the antagonistic compounds, *trans*-anethole successfully counteracted the antagonistic effect, restoring the LD_50_ values comparable to that of individual thymol.

**Figure 4:**
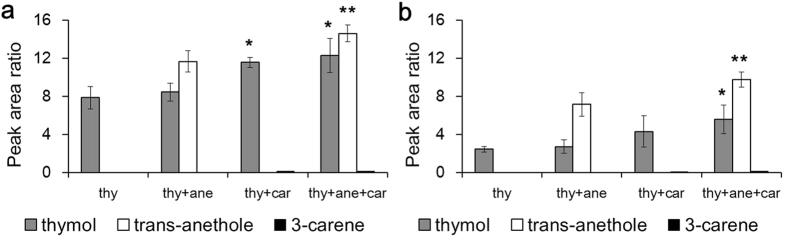
Penetration analyses of thymol and the mixtures with selected compounds *in vivo* in 1 h (**a**) and 2 h (**b**) post-treatment. In tertiary mixture, 3-carene significantly enhanced the penetration of thymol (*P = 0.01 (1 h) and 0.04 (2 h), respectively, compared to that of thymol alone) and *trans*-anethole (**P = 0.02 (1 h) and 0.04 (2 h), respectively, compared to the thymol + *trans*-anethole mixture).

That the mechanism of synergy for *trans*-anethole differed to that for the other boosting agents was suggested not only from the results of the bioassay and contact angle measurements, but also from the analysis of *in vivo* penetration. Recovery of thymol from larval extracts was the same between applications of individual thymol and the thymol + *trans*-anethole mixture, whereas internal thymol was significantly increased when thymol was blended with 3-carene (*P* = 0.03, Fig. 4a). In the tertiary mixture, 3-carene also significantly enhanced the penetration of *trans*-anethole (*P* = 0.02 and 0.04, respectively, at 1 h and 2 h observations). At 2 h post-administration, the peak area ratios of the compounds decreased substantially, suggesting rapid detoxicative metabolism and/or excretion. Although the peak area ratio of the binary mixture of thymol and 3-carene (4.3 ± 1.6) was greater than that for individual thymol (2.4 ± 0.3), they did not differ statistically; only the tertiary mixture showed a statistical difference (*P* = 0.04, 5.6 ± 1.5). Since there was no difference between individual thymol and the thymol + *trans*-anethole mixture at either time of observation, we suggest that *trans*-anethole affects neither the penetration nor the metabolism of thymol. No apparent metabolites or intermediate compounds were detected in the present study.

## Discussion

Most insecticide and antibiotic resistance results from either a loss of sensitivity at the target site or elevated detoxicative enzyme activity, and as resistance can be mitigated or delayed by rotating insecticides with different modes-of-action or in combination with enzyme inhibitors^23^,^24^,^25^, it has been a general assumption that synergy in botanical insecticides would be based on similar mechanisms. From pharmaceutical studies, synergistic mechanisms in multi-drug applications are proposed based on activation of multiple-target sites, interactions with resistance mechanisms, or pharmacokinetic effects that improve solubility^26^,^27^. Although pharmacokinetic effects have not been explored much for botanical insecticides, the adjuvant effect *via* enhanced permeability for essential oil constituents has been reported for antimicrobial activity and drug delivery systems in mammalian skins^2^,^28^.

In the present study, we tested the synergistic activity of plant essential oil constituents to confirm the penetration-enhancing effect through the insect integument to evaluate how common these effects are in terms of insecticidal activity. Our previous study showed that camphor was more potent than 1,8-cineole when those compounds were delivered into the larvae *via* injection^21^. For binary mixtures of camphor and 1,8-cineole, a ratio favoring camphor was less toxic than combinations with lower proportions of it (Table 1). This decreased toxicity was due to the limited solubility of camphor, and GC-MS analysis confirmed that the combination producing higher toxicity had a greater degree of penetration of camphor through the integument (Fig. 2). Thus the *in vitro* investigation confirms the presence of a penetration-enhancing effect in the synergistic combination as the main mechanism of synergy.

Among the tested compounds, several including eugenol, *trans*-cinnamaldehyde, camphor and borneol showed relatively high internal toxicity although their topical toxicity was not notable (Table 2). Those compounds were reported to exhibit strong activity in many insect and arthropod species as well as microorganisms^29^,^30^,^31^,^32^,^33^,^34^. Those compounds showed either relatively higher contact angles or rapid recrystallization on a treated surface, indicative of limited spreadability or solubility. Several factors such as application method, carrier solvent, membrane composition of target organism, and body size, can affect the bioactivity of compounds, and more specifically, physico-chemical properties may be in part responsible for observed interspecific differences in toxicity.

Although GC-MS analysis verified the penetration-enhancing effect in our study, it is a rather time-consuming process. A convenient diagnostic method is required to assess the penetration-enhancement of synergistic combinations, and we found that divided topical administration of the synergistic compounds can easily distinguish the combination effect, since it can minimize the interactions between them. A previous study using radio-labelled thymol showed minimal lateral movement of the compound across the integument of the cabbage looper following topical administration^35^, and our divided application method should have minimized the overlap of separately applied compounds. For example, for those combination ratios which did not produce any recrystallization of camphor due to increased solubility of the mixture, in the divided application a white residue of camphor was found on the spot where camphor was individually applied, because its solubility-increasing effect was eliminated.

Since many plant essential oil constituents are known to possess multiple modes-of-action^13^,^36^,^37^,^38^,^39^, synergistic activity observed has long been speculated to be obtained *via* complex effects in several targets or as a consequence of a different mechanism being targeted^2^. However, despite this complexity in their modes-of-action, the synergistic or antagonistic effects in essential oil-based insecticides seem to be quite simple; it is a question of how effectively the toxicant can be spread on the wax layer of the insect integument.

Contact angles of the mixtures on a beeswax layer showed direct correlation to toxicity, indicating that enhancing efficacy requires a higher affinity to the wax layer. However, several synergistic combinations with camphor failed to follow this general trend, as both contact angles of the mixtures as well as their toxicity increased simultaneously. As shown with 1,8-cineole and camphor, not only the spreadability on a wax layer but also the solubility of the compound can be important for toxicity, and no recrystallization of camphor was observed in those high mortality/contact angle combinations. Furthermore, the lower contact angle of an individual compound does not always guarantee an increase in toxicity or decrease in contact angles of the mixture. For example, individual fenchone had a relatively low contact angle, but its mixture with thymol, carvacrol or α-terpineol failed to show any significant change in contact angle, nor in the toxicity of those mixtures.

It is noteworthy that most of the synergistic as well as antagonistic interactions among the 39 tested compounds were identified as penetration-related effects. For example, 6 synergistic and 11 antagonistic combinations were identified with thymol, and the increased or decreased mortality in 16 out of 17 interactions (94.1%) of the mixture were eliminated *via* divided application. Likewise, more than 75% of interactions in carvacrol and α-terpineol were identified as penetration-related. Among those interactions, it is notable that *trans*-anethole was the only common denominator compound which showed increased mortality in divided applications. Other compounds which showed synergy in divided application including α-terpineol (to carvacrol), terpinen-4-ol and methyl salicylate (to α-terpineol) had relatively similar topical toxicity to their individual LD_50_ values, suggesting that the high toxicity in divided applications might simply come from their own toxicity, not because of any biochemical synergy.

It is clear that *trans*-anethole does not affect the penetration of thymol based on the GC-MS analysis *in vivo*, while 3-carene significantly enhances the penetration of both thymol and *trans*-anethole in the tertiary mixture (Fig. 4). Since *trans*-anethole does not affect the metabolism of thymol in the cabbage looper^19^, this observation suggests that those two compounds work independently *via* different synergistic mechanisms, internally (presumably by different modes-of-action) and externally (by increased penetration). Moreover, the toxicity response to thymol and *trans*-anethole support their different modes-of-action: while the larvae treated with thymol showed slowed movements leading to flaccid paralysis, *trans*-anethole administration produced unique tremors at the both ends, followed by paralysis (see Supplementary Video). However, the tertiary mixture with *trans*-anethole and 3-carene failed to enhance the toxicity of thymol any further (Table 4). Typically, botanical insecticides are formulated as water-based emulsions, and water has a negative impact on spreadability especially on a waxy surface. Based on the present study, given that increases in contact angle negatively impact topical toxicity, careful design of the surfactant, solvent and adjuvant system can be crucial to enhancing insecticide efficacy. Moreover, our observations may provide the impetus for comparable studies in mammalian systems, given the breadth of proposed therapeutic and antimicrobial properties of essential oils and/or their constituent terpenoids.

## Materials and Methods

### Insect maintenance

Eggs of the cabbage looper, *Trichoplusia ni*, were obtained from the Insect Production Service, Great Lakes Forestry Centre, Natural Resources Canada, Sault Ste. Marie, ON, Canada. Insects were reared on a pinto bean-based artificial diet in a growth chamber (25 ± 2 °C, 60% RH, and 16:8 h LD photoperiod).

### Bioassays

To examine the contact toxicity of individual compounds and their mixtures, a topical assay was conducted^21^. A group of ten 3rd instar larvae received 1 μL of test solution dissolved in acetone.

To evaluate the penetration of test compound combinations, a divided application assay was designed, and the mortality was compared to that of the mixture. Each of the test compounds in a binary mixture was dissolved in acetone separately at double the concentration in the mixture, and 0.5 μL of one solution was topically applied on the underside of the thorax, and another 0.5 μL on the dorsal surface of the abdomen of the third instar larva. Preliminary study showed no difference in mortality when the positions of the two compounds were switched.

To assess the internal toxicity of the test compounds, an injection assay was conducted using 5th instar larvae, introducing the compounds directly into the hemocoel close to the ventral nerve cord^21^. Each of a group of ten larvae was injected with 1 μL of 50% (w/v) solution using a microneedle under an optical microscope. Acetone was used as the negative (carrier) control in all bioassays, and it did not produce any mortality. All bioassays were repeated three times, and mortality was recorded after 24 h.

### Test compounds

To identify synergy between 1,8-cineole and camphor within a range of ratios, a series of binary mixtures was prepared, and their LD_50_ and LD_95_ values were determined *via* topical assay. At LD_95_ levels for each mixture, a divided application was performed and mortality was compared to that of the mixture as well as the individual compounds at their equivalent amounts.

Contact toxicity of 39 compounds was assessed *via* topical assay in 3rd instar larvae, and their internal toxicity was evaluated in 5th instar larvae by injecting them at 500 μg/insect. Three of the most toxic compounds based on topical toxicity (group A: thymol, carvacrol and α-terpineol) and another three which were considered potentially toxic (mildly or weakly toxic topically, but internally as toxic as the first three, group B: eugenol, *trans*-cinnamaldehyde and camphor) were chosen for further study.

The mixture effects of group A compounds at their LD_50_ doses were examined by mixing them with other compounds (1:1, w:w) in the topical assay, and mortality was compared to that in divided application assay to evaluate the penetration effect. As for the group B compounds, the combination effect was evaluated at the equal-toxic level (LD_20_ + LD_20_). Divided application was not performed for the group B mixtures. Lastly, the interactions of thymol and other selected compounds were examined *via* topical application in their binary or tertiary mixtures.

### Measurement of contact angles and physical property determination

Contact angles of the individual compounds and their mixtures were measured to examine affinities to a cuticular layer using a beeswax layer as a surrogate^21^. Three μL aliquots of test compounds dissolved in acetone (50%, v/v) were applied on the surface and the droplets photographed using a Dino-Lite digital microscope (AM4113T, AnMo Electronics Corp., New Taipei City, Taiwan). Contact angles were analyzed by using DinoCapture 2.0 software (ver. 1.5.14.G), and the process was repeated five times.

Four of the parameters commonly used in quantitative structure-activity relationship (QSAR) research were chosen. Octanol-water partition coefficient and surface tension were calculated using ACD/ChemSketch 2012 software (ver. 14.01), and solvent accessible surface area as well as polar surface area were determined from a chemicalize.org^beta^ database.

### Sample preparation for chemical analyses of cuticular penetration

The penetration of individual 1,8-cineole, camphor and mixtures thereof through the insect integument was investigated *in vitro*. A section of insect integument was prepared by decapitating a 5th instar larva and removing major organs and debris by thorough rinsing with deionized water. The layer was then mounted in a Franz cell diffusion system (ID 5 mm, 5 mL of receptor chamber, PermeGear, Inc., Hellertown, PA, USA) filled with HPLC grade n-hexanes^40^. A series of test solutions in different mixing ratios was prepared, and 1 μL of each solution (50% in acetone, w/v) was topically administrated on the upper surface of the preparation. The opening was covered with aluminum foil to minimize evaporation, and the receiver solution was carefully retrieved after 1 h of constant stirring with a Teflon-coated magnetic bar. Each treatment had ten larvae, and the test was repeated three times from different cohorts of cabbage loopers (See Supplementary Fig. S1 for details)

Potential change in penetration of thymol by the two synergizing compounds, *trans*-anethole and 3-carene, in a binary or tertiary mixture was examined by *in vivo* analysis following a previously reported method^21^ with slight modification. Test solutions were prepared at the equivalent amount of each compound in the LD_50_ dose of the tertiary mixture (LD_50_ = 37.6 μg/insect, ratio = 1:1:1) for thymol, thymol + *trans*-anethole, thymol + 3-carene, and thymol + *trans*-anethole + 3-carene. Each of twenty 3rd instar larvae received 1 μL of test solution and was transferred into a separate 4 mL glass vial with a loosely fitted cap. After 1 and 2 h of incubation, each larva was rinsed twice with n-hexanes (300 μL/larva each) for 1 min by gently shaking the vial. The solvent was removed and dried, and then twenty larvae were collected and transferred into a glass tissue homogenizer. One mL of n-hexane was added into the homogenizer, and the larvae were ground with ten strokes. The homogenate was immediately transferred into a clean vial using a glass pipette, and the pellet and the homogenizer were rinsed twice with another 2.5 mL of n-hexane each, and the rinse solution was combined with the homogenate. The test was repeated three times for each treatment, and the extracts were kept in a freezer until analyzed to minimize loss of compounds *via* evaporation.

The extraction method was previously validated through quantitative analysis of the larval extract and external rinse solution with n-hexanes^21^. We therefore did not repeat the quantification in the present study, but instead directly compared peak area ratios of each compound to that of internal standards as mentioned below.

### GC-MS analysis

Samples of individual 1,8-cineole and camphor and their binary mixtures obtained *in vitro* were analyzed by an Agilent 7890/5975 C GC-MS (Agilent Technologies Canada Inc., Ottawa, ON, Canada) fitted with a J&W DB-wax fused-silica column (30 m × 0.25 mm ID, 0.25 μm thickness) in electron ionization mode by a liquid injection mode. The injection was performed in a split mode (3:1) with a volume of 1.0 μL. The oven temperature was set at 40 °C for 3 min, increased to 240 °C with an increase of 25 °C/min, and held for 9 min. The total run time was 20 min, and helium was used as a carrier with 0.9 mL/min of flow. α-Pinene (retention time of 4.09 min) stock solution in n-hexanes was prepared for an internal standard, and each of the receiver solution containing individual 1,8-cineole (5.99 min), camphor (8.03 min) or their mixture was spiked with the internal standard solution (1 ug/mL) immediately before the analysis. The result was expressed as the peak area ratio between test compounds/α-pinene.

As for the *in vivo* extracts of individual thymol and its combinations with other selected compounds, the analysis was conducted on an Agilent 6890 N/5975 GC-MS fitted with a DB-wax column. The initial oven temperature was 50 °C for 3 min, with an increase at 30 °C/min to 230 °C, and the total run time was 14 min. Since α-pinene had a similar retention time to 3-carene in a preliminary validation test, *p*-cymene (5.35 min) was selected as an internal standard to evaluate the penetration of thymol (9.32 min), *trans*-anethole (8.14 min), 3-carene (4.17 min) and their mixture.

### Statistics

Probit analysis was used to determine LD_50_ values, and differences in mortality and penetration were determined by one-way ANOVA with Tukey’s test post hoc using StatPlus 2009 software (AnalystSoft, Alexandria, VA, USA). Interactions between 1,8-cineole and camphor as well as thymol and selected compounds were determined using the Hewlett and Plackett method^41^, and among the 39 compounds as per Hummelbrunner and Isman^42^ (See Supplementary Methods).

## Additional Information

**How to cite this article**: Tak, J.-H. and Isman, M. B. Penetration-enhancement underlies synergy of plant essential oil terpenoids as insecticides in the cabbage looper, *Trichoplusia ni. Sci. Rep.* **7**, 42432; doi: 10.1038/srep42432 (2017).

**Publisher's note:** Springer Nature remains neutral with regard to jurisdictional claims in published maps and institutional affiliations.

## Supplementary Material

Supplementary Information

Supplementary Video 1

## Figures and Tables

**Table 1:** Insecticidal activity of 1,8-cineole and camphor mixture in different ratio in third instar larvae of the cabbage looper.

ratio^a^	obs D_50_^b^	(95% CL^c^)	exp LD_50_^d^	R^e^	Mortality at LD_95_ of the mixture (%±SE)^f^			
					1,8-cineole alone	camphor alone	mixture application	divided application
100:0	264.9	237.8–294.9			96.7 ± 3.3			
90:10	205.0	170.9–235.2	280.8	1.4 (additive)	80.0 ± 5.8^ab^	0.0 ± 0.0^c^	83.3 ± 3.3^a^	63.3 ± 3.3^b^
75:25	194.1	167.8–217.7	304.7	1.6 (synergistic)	40.0 ± 11.5^ab^	6.7 ± 3.3^c^	76.7 ± 6.7^a^	23.3 ± 8.8^b^
60:40	186.9	151.2–215.8	328.6	1.8 (synergistic)	53.3 ± 8.8^b^	6.7 ± 3.3^c^	93.3 ± 6.7^a^	40.0 ± 5.8^b^
50:50	234.0	207.4–273.6	344.5	1.5 (synergistic)	46.7 ± 8.8^b^	10.0 ± 5.8^b^	96.7 ± 3.3^a^	46.7 ± 14.5^b^
40:60	236.1	202.9–268.6	360.4	1.5 (synergistic)	50.0 ± 5.8^b^	33.3 ± 3.3^b^	86.7 ± 7.7^a^	50.0 ± 5.8^b^
25:75	290.4	244.5–340.1	384.3	1.3 (additive)	20.0 ± 10.0^c^	63.3 ± 3.3^b^	93.3 ± 3.3^a^	76.7 ± 3.3^ab^
10:90	346.4	303.8–396.8	408.2	1.2 (additive)	0.0 ± 0.0^c^	86.7 ± 3.3^ab^	96.7 ± 3.3^a^	73.3 ± 8.8^b^
0:100	424.1	378.2–480.7				86.7 ± 6.7		

^a^Mixing ratio of (1,8-cineole : camphor).

^b^Observed LD_50_ values, μg/insect.

^c^Confidence limit.

^d^Expected LD_50_ values from Hewlett & Plackett model, μg/insect.

^e^Synergy ratio = (expected LD_50_) ÷(observed LD_50_), defined as synergistic when R > 1.5.

^f^Mortality (% ± SE) of individual 1,8-cineole and camphor at the equivalent amount in LD_95_ of the mixture at specific blending ratios, and *via* mixture and divided applications. Values with the same letter within a column do not differ significantly (Tukey HSD test, *P* = 0.05).

**Table 2:** Insecticidal activity of test compounds *via* topical application (3rd) and *via* injection (5th) in cabbage looper larvae and their contact angles on beeswax layer.

compound	LD_50_^a^	95% CL	Slope ± SE	injection mortality^b^	contact angle (° ± SE)
thymol	25.7	22.0–29.4	3.9 ± 0.5	83.3 ± 3.3	35.9 ± 0.6
carvacrol	36.3	28.3–46.6	4.4 ± 0.8	86.7 ± 3.3	35.0 ± 0.4
α-terpineol	67.9	59.9–77.5	4.1 ± 0.5	83.3 ± 8.8	31.7 ± 1.1
terpinen-4-ol	87.1	79.3–94.9	6.4 ± 0.8	80.0 ± 5.8	29.3 ± 0.8
methyl salicylate	87.8	71.1–105.8	2.9 ± 0.4	26.7 ± 12.0	39.4 ± 0.8
*trans*-anethole	91.8	81.1–104.4	4.2 ± 0.5	50.0 ± 5.8	34.3 ± 0.5
citral	97.4	80.8–113.2	3.3 ± 0.4	26.7 ± 6.7	31.5 ± 1.5
geranic acid	99.2	83.8–115.5	2.9 ± 0.3	20.0 ± 0.0	28.8 ± 0.7
4-nonanone	107.0	95.7–118.7	5.9 ± 0.8	73.3 ± 8.8	22.3 ± 0.8
*trans*-cinnamaldehyde	119.5	70.7–188.4	3.1 ± 0.7	80.0 ± 5.8	42.3 ± 0.3
eugenol	137.0	119.9–156.2	5.2 ± 0.7	90.0 ± 5.8	39.6 ± 0.6
linalool	141.1	125.0–157.6	5.2 ± 0.7	33.3 ± 3.3	25.0 ± 1.1
carvone	142.1	108.7–187.6	4.8 ± 1.1	33.3 ± 8.8	28.9 ± 1.6
geraniol	151.8	124.8–182.3	2.4 ± 0.3	20.0 ± 5.8	32.2 ± 0.8
menthol	163.4	147.6–182.3	6.1 ± 0.8	50.0 ± 5.8	29.6 ± 0.9
verbenone	175.3	153.0–203.5	3.5 ± 0.4	70.0 ± 0.0	38.3 ± 0.8
perillaldehyde	183.0	130.0–265.6	4.2 ± 0.9	60.0 ± 5.8	34.4 ± 1.1
menthone	209.4	156.2–262.1	8.1 ± 2.0	43.3 ± 3.3	24.0 ± 0.9
carveol	210.1	185.8–240.5	3.9 ± 0.5	36.7 ± 8.8	33.0 ± 1.2
bornyl acetate	264.1	234.6–300.4	4.5 ± 0.6	33.3 ± 6.7	25.2 ± 0.5
1,8-cineole	278.9	256.0–302.8	8.5 ± 1.1	46.7 ± 3.3	18.6 ± 0.4
citronellal	282.6	254.9–316.9	6.4 ± 0.9	26.7 ± 6.7	30.0 ± 0.9
γ-terpinene	298.8	274.6–326.2	6.2 ± 0.8	23.3 ± 3.3	17.5 ± 0.3
linalool oxide	315.3	282.2–353.4	5.3 ± 0.7	13.3 ± 6.7	30.9 ± 1.0
limonene	318.6	286.3–358.6	5.6 ± 0.8	30.0 ± 5.8	19.2 ± 0.8
α-pinene	335.7	302.3–373.3	5.6 ± 0.7	10.0 ± 0.0	16.5 ± 0.8
α-terpenene	362.1	328.0–400.4	6.3 ± 0.8	13.3 ± 8.8	16.3 ± 0.6
fenchone	382.6	319.3–450.3	3.5 ± 0.5	16.7 ± 3.3	21.0 ± 0.5
3-carene	425.6	370.9–501.9	3.7 ± 0.5	6.7 ± 3.3	14.6 ± 0.5
geranyl acetate	430.2	370.1–513.7	2.5 ± 0.3	6.7 ± 3.3	33.4 ± 0.6
*p*-cymene	435.0	392.1–491.7	6.1 ± 0.9	46.7 ± 3.3	17.8 ± 0.4
anisaldehyde	453.9	384.3–543.8	2.9 ± 0.4	10.0 ± 5.8	43.1 ± 0.5
β-pinene	481.0	412.2–554.6	3.4 ± 0.4	43.3 ± 8.8	17.1 ± 0.6
camphor	602.6	543.8–671.0	5.8 ± 0.8	76.7 ± 6.7	30.0 ± 0.7
linalyl acetate	658.1	538.4–855.2	2.3 ± 0.4	0.0 ± 0.0	28.0 ± 0.4
borneol	759.7	614.6–1097.9	2.7 ± 0.6	80.0 ± 5.8	29.2 ± 0.5
isoeugenol	859.4	735.7–1061.7	3.0 ± 0.6	26.7 ± 3.3	46.8 ± 1.0
camphene	>1000			6.7 ± 3.3	16.8 ± 0.2
caryophyllene oxide	>1000			3.3 ± 3.3	28.5 ± 0.4

^a^LD_50_ (μg/insect) *via* topical administration in 3rd instar larvae.

^b^Mortality (% ± SE) *via* injection assay in 5th instar larvae.

**Table 3:** List of interactions in binary mixtures.

Compound	#Of synergistic interaction	Most synergistic compound	#Of antagonistic interaction	Most antagonistic compound
thymol	6 (1)^a^	3-carene (87%)^b^	11	caryophyllene oxide (3%)
carvacrol	9 (2)	trans-anethole (100%)	6	camphor, caryophyllene oxide, verbenone (7%)
α-terpineol	10 (3)	terpinen-4-ol (100%)	2	camphene, *trans*-cinnamaldehyde (13%)
eugenol	25	3-carene, *p*-cymene, limonene, β-pinene, α-terpenene, γ-terpinene (100%)	5	geraniol, methyl salicylate, verbenone (17%)
*trans*-cinnamaldehyde	23	camphene, 3-carene, caryophyllene oxide, citronellal, limonene, menthone (100%)	11	isoeugenol, methyl salicylate (13%)
camphor	30	caryophyllene oxide, citronellal, menthol, 4-nonanone (100%)	0	

^a^Number of compounds also exhibiting synergistic interactions in the divided application.

^b^%mortality of the binary mixture in topical assay against 3rd instar larvae of the cabbage looper.

**Table 4:** Insecticidal activities of mixtures of thymol and selected compounds.

test compounds (w/w)	LD_50_^a^	95% CL^b^	Expected LD_50_	contact angle (°)	thymol amount^c^	change (%)^d^
thymol	24.9	22.0–28.2		34.4 ± 0.7	24.9	
+*trans*-anethole (1:1)	30.4	26.6–34.6	58.4	35.0 ± 0.8	15.2	−39.0
+3-carene (1:1)	36.0	32.5–39.8	225.3	27.5 ± 0.6	18.0	−27.7
+α-terpinene (1:1)	36.0	31.8–40.9	193.5	26.9 ± 0.7	18.0	−27.7
+anisaldehyde (1:1)	234.2	207.7–266.7	239.4	45.1 ± 0.5	117.1	370.3
+geranyl acetate (1:1)	105.8	64.4–171.6	227.6	40.1 ± 0.6	52.9	112.5
+*trans*-anethole + 3-carene (1:1:1)	37.6	33.1–42.8	179.0	31.8 ± 0.8	12.5	−49.6
+trans-anethole + α-terpinene (1:1:1)	43.4	38.4–49.0	158.0	29.2 ± 0.6	14.5	−41.9
+3-carene + α-terpinene (1:1:1)	58.3	52.7–64.5	268.2	25.1 ± 0.4	19.4	−21.9
+*trans*-anethole + anisaldehyde (1:1:1)	106.7	79.1–151.4	188.3	41.6 ± 0.8	35.6	42.9
+trans-anethole + geranyl acetate (1:1:1)	65.3	43.6–97.7	180.5	39.0 ± 0.4	21.8	−12.6

^a^μg/insect.

^b^Confidence limit.

^c^μg of thymol in LD_50_ dose of each treatment.

^d^% change of thymol amount compared to the individual LD_50_.
