# Supplementary material for: Penetration-enhancement underlies synergy of plant essential oil terpenoids as insecticides in the cabbage looper, Trichoplusia ni
Source: Sci Rep. 2017 Feb 9;7:42432. doi: 10.1038/srep42432 (PMC5299433; doi:10.1038/srep42432)
Supplement: Supplementary Information [file srep42432-s1.pdf]

Supplementary Information of

Penetration-enhancement underlies synergy of plant essential oil terpenoids as insecticides in the cabbage looper, *Trichoplusia ni*

Jun-Hyung Tak* and Murray B. Isman

Faculty of Land and Food Systems, University of BritishColumbia, Vancouver, BC, Canada

* To whom correspondence should be addressed. E-mail: saturnpg7@yahoo.com

Supplementary Methods

Determination of interactions using LD_{50} values of individual compounds

Synergistic interactions in the toxicity between 1,8-cineole and camphor as well as thymol and selected compounds were determined using the Hewlett and Plackett method[40]. An expected LD_{50} value in a mixture was calculated from an equation;

$$(\text{expected } LD_{50}) = (a \times LD_{50(A)}) + (b \times LD_{50(B)})$$

where a, b are the concentration of compound A and B, with $LD_{50(A)}$, $LD_{50(B)}$ are the LD_{50} values of individual compounds. The synergy ratio (SR) of the binary mixture was calculated by an

equation;

SR = (expected LD_{50}) / (observed LD_{50})

The interaction between the compounds were determined as synergistic (where SR ≥ 1.5), additive (1.5 > SR ≥ 0.5), or antagonistic (SR < 0.5).

Determination of interactions using observed mortality

Interactions among the 39 compounds were determined as per Hummelbrunner and Isman[41]. Expected mortalities were calculated by the formula;

(expected mortality) = $O_a + O_b(1 - O_a)$

where *Oa* and *Ob* are the observed mortality of each compound.

The interactions were determined as synergistic, additive or antagonistic based on χ^2 values;

χ^2 = [(observed mortality of mixture) – (expected mortality)]^2 / (expected mortality)

where χ^2 with df = 1 and $a = 0.05$ is 3.84, and it was determined as synergistic when $\chi^2 > 3.84$ with observed mortality being higher than expected, antagonistic when $\chi^2 > 3.84$ with observed < expected, or additive when $\chi^2 < 3.84$.

As for the three most toxic compounds, thymol, carvacrol and α-terpineol, their interactions were further verified *via* Student's t-test by comparing the mean mortality in topical assay of the mixture and divided application to those of individual compounds ($P = 0.05$).

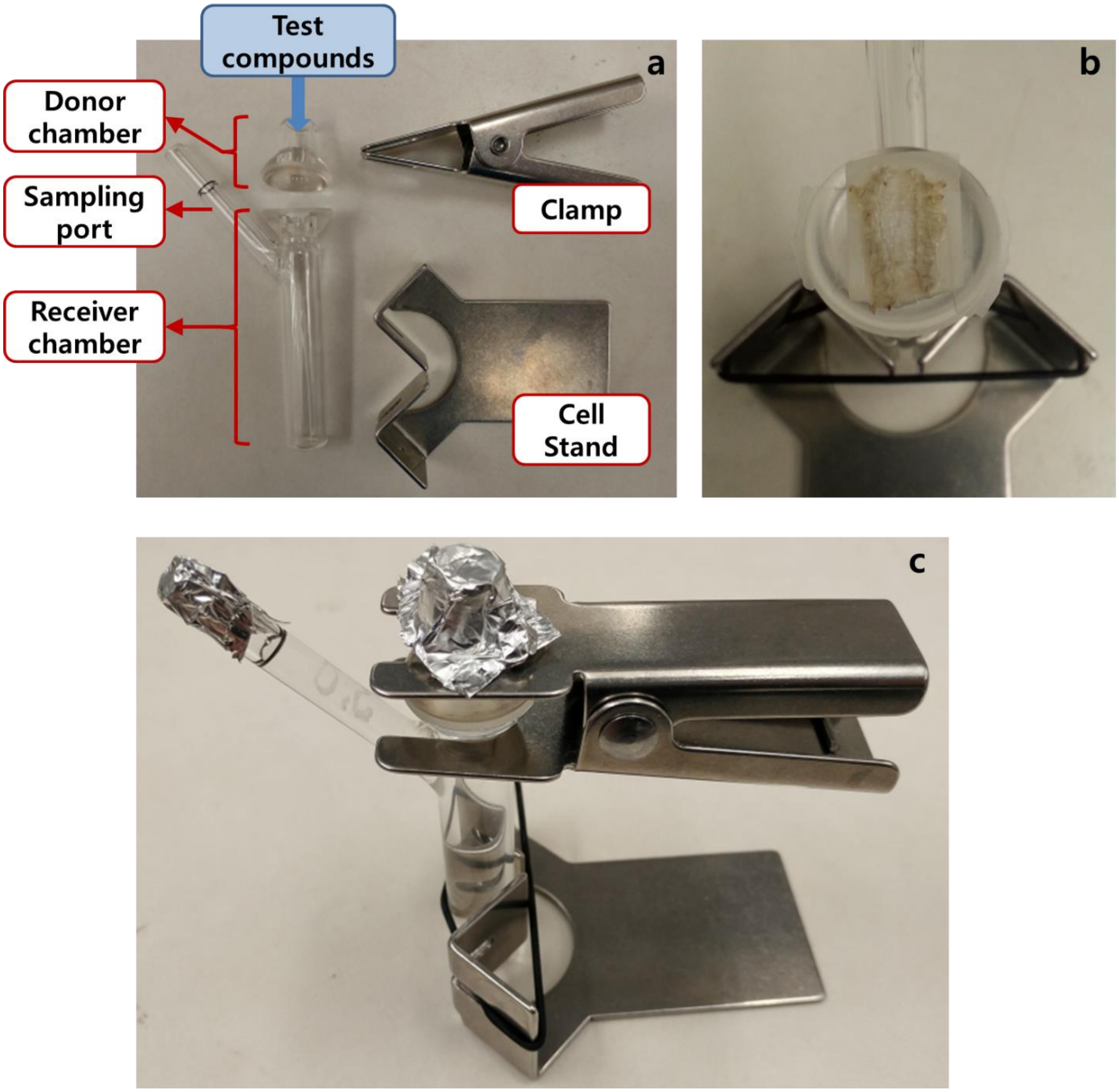

Supplementary Figure S1. A description of Franz cell diffuser system used for *in vitro* penetration analysis. (a) The receiver chamber has a sampling port, with a volume of 5 mL. Test compound solution was applied through the opening of the donor chamber. (b) Between donor and receiver chambers, an insect integument (cuticle) from a 5th instar larvae of *T. ni* was mounted. (c) The donor chamber and sampling port were covered with an aluminum foil to minimize evaporation.

Supplementary Table S1. Physical properties of 39 compounds

functional group	compound	log P	surface tension (dyne/cm)	polar surface area (Å ^{2})	molecular surface area (Å ^{2})
acetate	bornyl acetate	3.60	32.6	26.30	358.22
	geranyl acetate	4.10	28.4	26.30	356.89
	linalyl acetate	3.83	27.7	26.30	355.73
acid	geranic acid	3.46	32.6	37.30	288.59
alcohol	borneol	2.71	36.2	20.23	294.00
	carveol	2.55	32.6	20.23	259.39
	geraniol	3.28	29.1	20.23	293.77
	linalool	3.28	28.2	20.23	292.74
	menthol	3.20	29.7	20.23	313.32
	terpinen-4-ol	2.99	33.0	20.23	293.01
	α-terpineol	2.79	33.2	20.23	293.93
aldehyde	anisaldehyde	1.70	37.2	26.30	209.97
	trans-cinnamaldehyde	2.12	38.9	17.07	194.07
	citral	3.17	27.1	17.07	278.04
	citronellal	3.48	26.6	17.07	304.29
	perillaldehyde	2.68	40.1	17.07	245.61
ether	*trans*-anethole	3.17	31.8	9.23	248.20
	1,8-cineole	2.82	32.4	9.23	301.71
	methyl salicylate	2.23	45.7	46.53	219.62
hydrocarbon	camphene	4.37	27.0	0.00	252.36
	3-carene	4.37	25.3	0.00	258.88
	p-cymene	4.02	28.5	0.00	260.74
	limonene	4.45	25.8	0.00	252.39
	α-pinene	4.37	25.3	0.00	255.47
	β-pinene	4.37	27.0	0.00	253.51
	α-terpenene	4.53	26.8	0.00	254.45
	γ-terpinene	4.36	26.8	0.00	254.45
ketone	camphor	2.13	31.5	17.07	284.20
	carvone	2.26	29.8	17.07	248.59
	fenchone	2.13	31.5	17.07	283.72
	menthone	2.63	27.2	17.07	301.18
	4-nonanone	3.03	26.5	17.07	304.55
	verbenone	1.97	29.4	17.07	253.17
oxide	caryophyllene oxide	4.57	32.5	12.53	400.78
	linalool oxide	1.56	39.9	20.23	292.74
phenol	carvacrol	3.28	34.9	20.23	271.38
	eugenol	2.20	36.5	29.46	257.78
	isoeugenol	2.45	38.9	29.46	258.92
	thymol	3.28	34.9	20.23	271.44

Supplementary Table S2. Insecticidal activity and contact angle of thymol at LD_{50} dose in the binary mixture with other compounds (w:w=1:1)

compound	mortality (% ± SE)[a]		exp	mixture		divided		contact angle
	mixture	divided	mort[b]	χ^2	note[c]	χ^2	note[c]	(° ± SE)
trans-anethole	80.0 ± 5.8	83.3 ± 3.3	56.7	9.57	S	12.51	S	34.6 ± 0.5
anisaldehyde	6.7 ± 3.3	53.3 ± 12.0	56.7	44.15	A	0.20		39.5 ± 1.4
borneol	23.3 ± 8.8	50.0 ± 15.3	56.7	19.64	A	0.79		35.4 ± 0.9
bornyl acetate	43.3 ± 12.0	40.0 ± 5.8	56.7	3.15		4.92	A (NS)	32.5 ± 0.5
camphene	16.7 ± 12.0	53.3 ± 3.3	56.7	28.27	A	0.20		25.1 ± 0.5
camphor	26.7 ± 6.7	36.7 ± 8.8	56.7	15.91	A	7.08	A (NS)	36.8 ± 1.1
3-carene	86.7 ± 6.7	40.0 ± 15.3	56.7	15.84	S	4.92	A (NS)	23.9 ± 0.7
carvacrol	83.3 ± 6.7	100.0 ± 0.0	67.7	3.59		15.36	S	38.3 ± 0.4
carveol	53.3 ± 8.8	50.0 ± 5.8	56.7	0.20		0.79		37.6 ± 0.5
carvone	20.0 ± 10.0	40.0 ± 5.8	56.7	23.75	A	4.92	A (NS)	38.0 ± 0.2
caryophyllene oxide	3.3 ± 3.3	40.0 ± 5.8	56.7	50.23	A	4.92	A (NS)	34.7 ± 0.5
1,8-cineole	53.3 ± 21.9	46.7 ± 8.8	56.7	0.20		1.78		27.9 ± 0.8
trans-cinnamaldehyde	6.7 ± 3.3	43.3 ± 8.8	56.7	44.15	A	3.15		37.6 ± 1.4
citral	36.7 ± 6.7	36.7 ± 6.7	56.7	7.08	A	7.08	A (NS)	33.6 ± 0.6
citronellal	40.0 ± 11.5	46.7 ± 16.7	56.7	4.92	A (NS)	1.78		33.7 ± 0.9
p-cymene	83.3 ± 3.3	56.7 ± 8.8	56.7	12.51	S	0.00		25.7 ± 1.4
eugenol	53.3 ± 8.8	73.3 ± 3.3	56.7	0.20		4.88	S	39.9 ± 0.4
fenchone	50.0 ± 11.5	43.3 ± 3.3	56.7	0.79		3.15		34.4 ± 0.6
geranic acid	50.0 ± 10.0	63.3 ± 12.0	56.7	0.79		0.78		38.4 ± 1.1
geraniol	73.3 ± 8.8	50.0 ± 10.0	56.7	4.88	S (NS)	0.79		32.9 ± 0.7
geranyl acetate	16.7 ± 3.3	40.0 ± 15.3	56.7	28.27	A	4.92	A (NS)	36.7 ± 0.6
isoeugenol	53.3 ± 21.9	63.3 ± 8.8	56.7	0.20		0.78		41.7 ± 0.4
limonene	83.3 ± 3.3	50.0 ± 10.0	56.7	12.51	S	0.79		25.9 ± 0.6
linalool	46.7 ± 8.8	50.0 ± 0.0	56.7	1.78		0.79		34.4 ± 0.5
linalool oxide	23.3 ± 3.3	53.3 ± 12.0	56.7	19.64	A	0.20		34.0 ± 1.0
linalyl acetate	53.3 ± 6.7	56.7 ± 13.3	56.7	0.20		0.00		33.2 ± 0.4
menthol	53.3 ± 3.3	53.3 ± 6.7	56.7	0.20		0.20		30.5 ± 0.9
menthone	50.0 ± 15.3	40.0 ± 5.8	56.7	0.79		4.92	A (NS)	30.0 ± 1.0
methyl salicylate	76.7 ± 8.8	60.0 ± 10.0	59.7	4.80	S (NS)	0.00		39.4 ± 0.8
4-nonanone	60.0 ± 5.8	53.3 ± 12.0	56.7	0.19		0.20		31.9 ± 0.6
perillaldehyde	56.7 ± 16.7	53.3 ± 12.0	56.7	0.00		0.20		38.8 ± 1.2
α-pinene	63.3 ± 8.8	63.3 ± 8.8	56.7	0.78		0.78		25.7 ± 1.0
β-pinene	63.3 ± 3.3	60.0 ± 5.8	56.7	0.78		0.19		25.2 ± 0.8
terpinen-4-ol	63.3 ± 3.3	46.7 ± 6.7	56.7	0.78		1.78		34.1 ± 0.2
α-terpenene	80.0 ± 5.8	56.7 ± 18.6	56.7	9.57	S	0.00		21.9 ± 0.6
γ-terpinene	80.0 ± 5.8	53.3 ± 6.7	56.7	9.57	S	0.20		25.7 ± 0.6
α-terpineol	73.3 ± 3.3	63.3 ± 3.3	58.6	3.73		0.39		36.3 ± 0.7
verbenone	13.3 ± 3.3	36.7 ± 12.0	56.7	33.17	A	7.08	A (NS)	36.6 ± 0.7

[a]% mortality observed from the bioassay in mixture and divided application

[b]expected mortality from the probit analysis of each compound and observed mortality of thymol (56.7% ± 3.3)

[c]when χ^2>3.84, S: synergy (observed mortality > expected mortality), A: antagonism (observed < expected). NS denotes being not significant in the mean's comparison *via* Student t-test (P = 0.05).

Supplementary Table S3. Insecticidal activity and contact angle of carvacrol at LD_{50} dose in the binary mixture with other compounds (w:w=1:1)

compound	mortality (% ± SE)[a]		exp	mixture		divided		contact angle
	mixture	divided	mort[b]	χ^2	note[c]	χ^2	note[c]	(°)
trans-anethole	100.0 ± 0.0	70.0 ± 11.5	59.0	28.53	S	7.49	S	38.8 ± 1.1
anisaldehyde	53.3 ± 3.3	73.3 ± 3.3	57.0	0.24		4.68	S (NS)	45.3 ± 1.9
borneol	13.3 ± 8.8	43.3 ± 3.3	57.0	33.45	A	3.28		33.4 ± 0.7
bornyl acetate	60.0 ± 5.8	43.3 ± 3.3	57.0	0.16		3.28		30.9 ± 0.9
camphene	33.3 ± 8.8	43.3 ± 14.5	57.0	9.83	A (NS)	3.28		25.2 ± 0.3
camphor	6.7 ± 6.7	33.3 ± 8.8	57.0	44.45	A	9.83	A (NS)	34.8 ± 0.8
3-carene	96.7 ± 3.3	73.3 ± 6.7	57.0	27.60	S	4.68	S (NS)	26.7 ± 0.7
carveol	70.0 ± 10.0	56.7 ± 3.3	57.0	2.96		0.00		37.6 ± 1.2
carvone	43.3 ± 8.8	66.7 ± 12.0	57.0	3.28		1.64		37.4 ± 1.3
caryophyllene oxide	6.7 ± 6.7	50.0 ± 5.8	57.0	44.45	A	0.86		34.0 ± 1.1
1,8-cineole	50.0 ± 10.0	43.3 ± 6.7	59.3	1.47		4.31	A (NS)	43.4 ± 1.0
trans-cinnamaldehyde	40.0 ± 11.5	66.7 ± 8.8	57.0	5.07	A (NS)	1.64		30.4 ± 0.9
citral	46.7 ± 17.6	36.7 ± 3.3	60.5	3.16		9.38	A (NS)	34.4 ± 1.4
citronellal	73.3 ± 13.3	33.3 ± 3.3	57.0	4.68	S (NS)	9.83	A	35.8 ± 0.7
p-cymene	93.3 ± 6.7	60.0 ± 5.8	57.0	23.16	S	0.16		29.2 ± 0.3
eugenol	63.3 ± 8.8	73.3 ± 12.0	58.1	0.48		4.01	S (NS)	43.0 ± 1.1
fenchone	46.7 ± 3.3	46.7 ± 3.3	57.0	1.87		1.87		33.5 ± 0.2
geranic acid	50.0 ± 10.0	63.3 ± 3.3	61.5	2.14		0.06		37.5 ± 0.9
geraniol	43.3 ± 20.3	20.0 ± 5.8	68.4	9.21	A (NS)	34.28	A	36.7 ± 1.2
geranyl acetate	16.7 ± 6.7	56.7 ± 3.3	57.0	28.54	A	0.00		38.5 ± 1.2
isoeugenol	50.0 ± 5.8	76.7 ± 3.3	57.0	0.86		6.79	S (NS)	44.6 ± 1.5
limonene	93.3 ± 3.3	36.7 ± 8.8	57.0	23.16	S	7.25	A (NS)	26.3 ± 1.0
linalool	80.0 ± 5.8	56.7 ± 8.8	57.0	9.28	S	0.00		31.8 ± 0.8
linalool oxide	16.7 ± 3.3	43.3 ± 3.3	57.0	28.54	A	3.28		34.8 ± 0.4
linalyl acetate	43.3 ± 18.6	50.0 ± 5.8	57.0	3.28		0.86		34.4 ± 1.1
menthol	63.3 ± 3.3	36.7 ± 3.3	57.0	0.70		7.25	A (NS)	34.3 ± 0.3
menthone	60.0 ± 5.8	50.0 ± 5.8	57.0	0.16		0.86		35.4 ± 0.8
methyl salicylate	63.3 ± 8.8	73.3 ± 12.0	61.8	0.04		2.16		40.1 ± 1.0
4-nonanone	60.0 ± 5.8	43.3 ± 3.3	57.0	0.16		3.28		33.3 ± 0.5
perillaldehyde	53.3 ± 3.3	40.0 ± 15.3	57.0	0.24		5.07	A (NS)	38.0 ± 0.7
α-pinene	73.3 ± 3.3	43.3 ± 3.3	57.0	4.68	S (NS)	3.28		21.1 ± 0.6
β-pinene	86.7 ± 8.8	56.7 ± 3.3	57.0	15.44	S	0.00		26.5 ± 1.2
terpinen-4-ol	83.3 ± 6.7	63.3 ± 6.7	57.0	12.17	S	0.70		35.3 ± 0.9
α-terpenene	83.3 ± 6.7	56.7 ± 3.3	57.0	12.17	S	0.00		29.1 ± 0.7
γ-terpinene	76.7 ± 14.5	50.0 ± 5.8	57.0	6.79	S (NS)	0.86		23.1 ± 0.5
α-terpineol	86.7 ± 6.7	86.7 ± 8.8	62.7	9.18	S	9.18	S	38.0 ± 1.2
verbenone	6.7 ± 6.7	56.7 ± 8.8	57.0	44.45	A	0.00		36.3 ± 1.5

[a]% mortality observed from the bioassay in mixture and divided application

[b]expected mortality from the probit analysis of each compound and observed mortality of carvacrol (56.7% ± 8.8)

[c]when $\chi^2 > 3.84$, S: synergy (observed mortality > expected mortality), A: antagonism (observed < expected). NS denotes being not significant in the mean's comparison *via* Student t-test ($P = 0.05$).

Supplementary Table S4. Insecticidal activity and contact angle of α-terpineol at LD_{50} dose in the binary mixture with other compounds (w:w=1:1)

compound	mortality (% ± SE)[a]		exp mort[b]	mixture		divided		contact angle
	mixture	divided		χ^2	note[c]	χ^2	note[c]	(°)
trans-anethole	96.7 ± 3.3	90.0 ± 5.8	54.4	32.84	S	23.30	S	33.0 ± 2.2
anisaldehyde	30.0 ± 10.0	33.3 ± 3.3	41.7	3.28	A (NS)	1.68		38.8 ± 1.0
borneol	36.7 ± 14.5	46.7 ± 6.7	41.7	0.61		0.59		33.7 ± 1.2
bornyl acetate	76.7 ± 12.0	63.3 ± 3.3	41.7	29.32	S (NS)	11.22	S (NS)	29.9 ± 0.8
camphene	13.3 ± 6.7	46.7 ± 6.7	41.7	19.30	A	0.59		25.8 ± 1.1
camphor	46.7 ± 14.5	46.7 ± 3.3	41.7	0.59		0.59		29.9 ± 0.6
3-carene	76.7 ± 3.3	56.7 ± 13.3	41.7	29.32	S (NS)	5.37	S (NS)	23.1 ± 0.6
carveol	66.7 ± 3.3	50.0 ± 20.0	42.9	13.17	S (NS)	1.18		27.1 ± 1.4
carvone	70.0 ± 10.0	60.0 ± 11.5	44.0	15.33	S (NS)	5.80	S (NS)	34.1 ± 0.8
caryophyllene oxide	40.0 ± 5.8	53.3 ± 12.0	41.7	0.07		3.25		32.4 ± 1.0
1,8-cineole	83.3 ± 8.8	36.7 ± 13.3	41.7	41.57	S	0.61		27.0 ± 0.6
trans-cinnamaldehyde	13.3 ± 8.8	46.7 ± 8.8	52.0	28.71	A	0.54		42.1 ± 1.5
citral	70.0 ± 5.8	63.3 ± 8.8	56.3	3.35		0.89		34.2 ± 0.7
citronellal	70.0 ± 10.0	50.0 ± 11.5	41.7	19.21	S (NS)	1.65		30.4 ± 1.3
p-cymene	86.7 ± 3.3	33.3 ± 12.0	41.7	48.49	S	1.68		26.6 ± 0.5
eugenol	53.3 ± 6.7	70.0 ± 10.0	45.1	1.49		13.70	S (NS)	38.2 ± 0.5
fenchone	60.0 ± 5.8	33.3 ± 8.8	41.7	8.03	S (NS)	1.68		34.5 ± 0.3
geranic acid	53.3 ± 6.7	56.7 ± 3.3	57.1	0.25		0.00		36.1 ± 0.3
geraniol	60.0 ± 20.8	50.0 ± 5.8	51.6	1.38		0.05		32.9 ± 1.0
geranyl acetate	83.3 ± 12.0	50.0 ± 10.0	42.7	38.75	S (NS)	1.26		32.9 ± 1.5
isoeugenol	23.3 ± 13.3	33.3 ± 12.0	41.7	8.09	A (NS)	1.68		38.4 ± 1.3
limonene	83.3 ± 3.3	43.3 ± 14.5	41.7	41.57	S	0.06		25.5 ± 0.6
linalool	76.7 ± 6.7	60.0 ± 15.3	43.6	25.15	S (NS)	6.20	S (NS)	31.0 ± 1.1
linalool oxide	70.0 ± 5.8	40.0 ± 5.8	41.7	19.21	S (NS)	0.07		31.6 ± 1.4
linalyl acetate	66.7 ± 3.3	33.3 ± 6.7	41.7	14.95	S (NS)	1.68		28.6 ± 0.9
menthol	80.0 ± 5.8	36.7 ± 3.3	41.7	35.18	S	0.61		26.3 ± 0.9
menthone	83.3 ± 6.7	63.3 ± 8.8	41.7	41.57	S	11.22	S (NS)	28.5 ± 0.4
methyl salicylate	93.3 ± 3.3	80.0 ± 5.8	57.5	22.36	S	8.82	S	38.3 ± 1.0
4-nonanone	93.3 ± 3.3	36.7 ± 12.0	45.8	49.28	S	1.83		29.3 ± 0.6
perillaldehyde	66.7 ± 8.8	56.7 ± 8.8	43.2	12.75	S (NS)	4.20	S (NS)	33.6 ± 1.3
α-pinene	73.3 ± 12.0	40.0 ± 11.5	41.7	24.00	S (NS)	0.07		23.4 ± 0.9
β-pinene	66.7 ± 3.3	40.0 ± 5.8	41.7	14.95	S (NS)	0.07		24.0 ± 0.6
terpinen-4-ol	100.0 ± 0.0	80.0 ± 5.8	50.4	48.71	S	17.33	S	31.8 ± 0.9
α-terpenene	83.3 ± 3.3	46.7 ± 12.0	41.7	41.57	S	0.59		23.4 ± 0.6
γ-terpinene	76.7 ± 8.8	46.7 ± 6.7	41.7	29.32	S (NS)	0.59		23.2 ± 0.2
verbenone	46.7 ± 12.0	66.7 ± 6.7	44.6	0.09		10.90	S (NS)	35.5 ± 1.0

[a]% mortality observed from the bioassay in mixture and divided application

[b]expected mortality from the probit analysis of each compound and observed mortality of α-terpineol (41.7% ± 9.5)

[c]when $\chi^2>3.84$, S: synergy (observed mortality > expected mortality), A: antagonism (observed < expected). NS denotes being not significant in the mean's comparison *via* Student t-test ($P = 0.05$).

Supplementary Table S5. Insecticidal activity of eugenol with other compounds

compound	LD_{20}[a]	ratio[b]	mortality (%) ± SE[c]	χ^2	note	contact angle (°)
trans-cinnamaldehyde	64.1	0.4	43.3 ± 8.8	0.28		37.9 ± 0.6
camphor	432.7	0.8	70.0 ± 5.8	22.50	S	38.8 ± 0.8
trans-anethole	58.0	0.4	43.3 ± 3.3	0.28		39.1 ± 1.0
anisaldehyde	235.3	0.7	36.7 ± 3.3	0.28		44.8 ± 0.6
borneol	372.4	0.8	36.7 ± 3.3	0.28		34.4 ± 0.3
bornyl acetate	171.7	0.6	96.7 ± 3.3	80.28	S	30.6 ± 0.7
camphene[d]	500.0	0.8	83.3 ± 6.7	46.94	S	24.3 ± 0.8
3-carene	251.7	0.7	100.0 ± 0.0	90.00	S	25.5 ± 0.7
carvacrol	23.4	0.2	50.0 ± 5.8	2.50		36.1 ± 1.3
carveol	127.8	0.6	70.0 ± 5.8	22.50	S	33.1 ± 0.6
carvone	95.0	0.5	26.7 ± 8.8	4.44	A	35.2 ± 0.8
caryophyllene oxide[d]	500.0	0.8	66.7 ± 3.3	17.78	S	36.7 ± 0.9
1,8-cineole	222.1	0.7	80.0 ± 5.8	40.00	S	28.6 ± 0.6
citral	54.0	0.4	40.0 ± 5.8	0.00		36.0 ± 1.3
citronellal	209.1	0.7	96.7 ± 3.3	80.28	S	30.4 ± 0.8
p-cymene	316.2	0.8	100.0 ± 0.0	90.00	S	28.6 ± 0.9
fenchone	221.4	0.7	86.7 ± 8.8	54.44	S	30.5 ± 0.4
geranic acid	50.7	0.3	53.3 ± 8.8	4.44	S	37.0 ± 1.1
geraniol	67.1	0.4	16.7 ± 8.8	13.61	A	37.3 ± 1.3
geranyl acetate	201.2	0.7	86.7 ± 8.8	54.44	S	36.4 ± 0.6
isoeugenol	446.9	0.8	20.0 ± 5.8	10.00	A	48.8 ± 1.1
limonene	225.6	0.7	100.0 ± 0.0	90.00	S	24.5 ± 1.2
linalool	97.1	0.5	60.0 ± 5.8	10.00	S	34.6 ± 1.3
linalool oxide	218.4	0.7	36.7 ± 12.0	0.28		38.0 ± 1.3
linalyl acetate	282.6	0.7	33.3 ± 13.3	1.11	A	39.4 ± 0.9
menthol	119.1	0.6	73.3 ± 3.3	27.78	S	32.5 ± 1.1
menthone	164.6	0.6	93.3 ± 6.7	71.11	S	30.1 ± 0.8
methyl salicylate	45.5	0.3	16.7 ± 12.0	13.61	A	37.8 ± 0.5
4-nonanone	77.2	0.4	86.7 ± 3.3	54.44	S	32.1 ± 0.8
perillaldehyde	115.2	0.5	63.3 ± 8.8	13.61	S	35.8 ± 1.5
α-pinene	238.0	0.7	96.7 ± 3.3	80.28	S	24.0 ± 0.4
β-pinene	273.1	0.7	100.0 ± 0.0	90.00	S	22.0 ± 0.8
terpinen-4-ol	64.4	0.4	66.7 ± 13.3	17.78	S	35.2 ± 0.6
α-terpenene	265.8	0.7	100.0 ± 0.0	90.00	S	27.2 ± 0.8
γ-terpinene	218.3	0.7	100.0 ± 0.0	90.00	S	24.3 ± 1.0
α-terpineol	42.5	0.3	60.0 ± 15.3	10.00	S	34.7 ± 1.6
thymol	15.7	0.1	56.7 ± 17.6	6.94	S	33.9 ± 0.7
verbenone	101.3	0.5	16.7 ± 3.3	13.61	A	37.4 ± 0.7

[a]LD_{20} value of each compound (μg/insect)

[b]mixing ratio of each compound in the binary mixture of eugenol (w:w)

[c]individual mortality of eugenol was 16.7% ± 3.3

[d]the amount of camphene and caryophyllene oxide was set to 500 μg

Supplementary Table S6. Insecticidal activity of *trans*-cinnamaldehyde with other compounds

compound	LD_{20}[a]	ratio[b]	mortality (%) ± SE[c]	χ^2	note	contact angle (°)
camphor	432.7	0.9	73.3 ± 3.3	27.78	S	38.1 ± 1.0
trans-anethole	58.0	0.5	33.3 ± 14.5	1.11		38.8 ± 0.8
anisaldehyde	235.3	0.8	16.7 ± 6.7	13.61	A	43.9 ± 0.9
borneol	372.4	0.9	36.7 ± 3.3	0.28		35.6 ± 0.9
bornyl acetate	171.7	0.7	93.3 ± 6.7	71.11	S	35.4 ± 1.0
camphene	500.0	0.9	100.0 ± 0.0	90.00	S	28.3 ± 0.5
3-carene	251.7	0.8	100.0 ± 0.0	90.00	S	25.5 ± 1.8
carvacrol	23.4	0.3	23.3 ± 18.6	6.94	A	36.4 ± 0.7
carveol	127.8	0.7	56.7 ± 8.8	6.94	S	38.3 ± 0.9
carvone	95.0	0.6	56.7 ± 12.0	6.94	S	40.5 ± 1.0
caryophyllene oxide	500.0	0.9	100.0 ± 0.0	90.00	S	38.9 ± 0.5
1,8-cineole	222.1	0.8	93.3 ± 3.3	71.11	S	28.2 ± 1.0
citral	54.0	0.5	26.7 ± 14.5	4.44	A	37.5 ± 0.5
citronellal	209.1	0.8	100.0 ± 0.0	90.00	S	36.9 ± 1.3
p-cymene	316.2	0.8	93.3 ± 6.7	71.11	S	29.4 ± 0.9
fenchone	221.4	0.8	93.3 ± 6.7	71.11	S	33.7 ± 0.8
geranic acid	50.7	0.4	70.0 ± 5.8	22.50	S	31.8 ± 1.1
geraniol	67.1	0.5	23.3 ± 12.0	6.94	A	37.6 ± 0.9
geranyl acetate	201.2	0.8	83.3 ± 8.8	46.94	S	39.2 ± 0.9
isoeugenol	446.9	0.9	13.3 ± 8.8	17.78	A	51.4 ± 2.6
limonene	225.6	0.8	100.0 ± 0.0	90.00	S	36.1 ± 0.9
linalool	97.1	0.6	10.0 ± 5.8	22.50	A	37.8 ± 0.7
linalool oxide	218.4	0.8	23.3 ± 8.8	6.94	A	39.6 ± 1.2
linalyl acetate	282.6	0.8	76.7 ± 8.8	33.61	S	36.8 ± 0.7
menthol	119.1	0.7	86.7 ± 8.8	54.44	S	31.7 ± 0.7
menthone	164.6	0.7	100.0 ± 0.0	90.00	S	35.1 ± 1.2
methyl salicylate	45.5	0.4	13.3 ± 6.7	17.78	A	37.0 ± 0.4
4-nonanone	77.2	0.5	96.7 ± 3.3	80.28	S	36.9 ± 0.5
perillaldehyde	115.2	0.6	90.0 ± 5.8	62.50	S	40.5 ± 0.9
α-pinene	238.0	0.8	63.3 ± 14.5	13.61	S	24.4 ± 1.1
β-pinene	273.1	0.8	93.3 ± 3.3	71.11	S	23.3 ± 1.8
terpinen-4-ol	64.4	0.5	26.7 ± 8.8	4.44	A	36.5 ± 0.6
α-terpenene	265.8	0.8	86.7 ± 3.3	54.44	S	31.1 ± 1.4
γ-terpinene	218.3	0.8	96.7 ± 3.3	80.28	S	28.2 ± 0.8
α-terpineol	42.5	0.4	30.0 ± 15.3	2.50		36.7 ± 1.3
thymol	15.7	0.2	20.0 ± 10.0	10.00	A	38.0 ± 0.6
verbenone	101.3	0.6	23.3 ± 8.8	6.94	A	39.1 ± 1.5

[a] LD_{20} value of each compound (μg/insect)

[b] mixing ratio of each compound in the binary mixture of *trans*-cinnamaldehyde (w:w)

[c] individual mortality of *trans*-cinnamaldehyde was 20.0% ± 5.8

[d] the amount of camphene and caryophyllene oxide was set to 500 μg

Supplementary Table S7. Insecticidal activity of camphor with other compounds

compound	LD_{20}[a]	ratio[b]	mortality (%) ± SE[c]	χ^2	note	contact angle (°)
trans-anethole	58.0	0.1	83.3 ± 3.3	46.94	S	30.3 ± 1.3
anisaldehyde	235.3	0.4	63.3 ± 3.3	13.61	S	42.4 ± 0.7
borneol	372.4	0.5	73.3 ± 8.8	27.78	S	43.1 ± 1.3
bornyl acetate	171.7	0.3	96.7 ± 3.3	80.28	S	32.6 ± 0.7
camphene	500.0	0.5	50.0 ± 5.8	2.50		52.4 ± 2.0
3-carene	251.7	0.4	96.7 ± 3.3	80.28	S	24.8 ± 0.3
carvacrol	23.4	0.1	30.0 ± 11.5	2.50		28.4 ± 1.7
carveol	127.8	0.2	90.0 ± 0.0	62.50	S	36.4 ± 0.5
carvone	95.0	0.2	96.7 ± 3.3	80.28	S	35.4 ± 1.2
caryophyllene oxide	500.0	0.5	100.0 ± 0.0	90.00	S	39.5 ± 0.9
1,8-cineole	222.1	0.3	96.7 ± 3.3	80.28	S	29.5 ± 0.5
citral	54.0	0.1	30.0 ± 10.0	2.50		30.8 ± 0.8
citronellal	209.1	0.3	100.0 ± 0.0	90.00	S	29.6 ± 0.2
p-cymene	316.2	0.4	86.7 ± 8.8	54.44	S	27.0 ± 1.0
fenchone	221.4	0.3	93.3 ± 3.3	71.11	S	28.3 ± 3.1
geranic acid	50.7	0.1	63.3 ± 8.8	13.61	S	31.4 ± 1.3
geraniol	67.1	0.1	56.7 ± 3.3	6.94	S	31.9 ± 0.9
geranyl acetate	201.2	0.3	90.0 ± 0.0	62.50	S	34.9 ± 0.5
isoeugenol	446.9	0.5	36.7 ± 6.7	0.28		45.3 ± 1.3
limonene	225.6	0.3	96.7 ± 3.3	80.28	S	31.2 ± 1.1
linalool	97.1	0.2	50.0 ± 5.8	2.50		32.8 ± 0.5
linalool oxide	218.4	0.3	73.3 ± 14.5	27.78	S	30.9 ± 1.2
linalyl acetate	282.6	0.4	66.7 ± 14.5	17.78	S	33.3 ± 1.0
menthol	119.1	0.2	100.0 ± 0.0	90.00	S	28.6 ± 0.6
menthone	164.6	0.3	96.7 ± 3.3	80.28	S	29.0 ± 1.0
methyl salicylate	45.5	0.1	73.3 ± 3.3	27.78	S	33.2 ± 0.9
4-nonanone	77.2	0.2	100.0 ± 0.0	90.00	S	26.1 ± 0.6
perillaldehyde	115.2	0.2	90.0 ± 0.0	62.50	S	32.5 ± 1.3
α-pinene	238.0	0.4	86.7 ± 3.3	54.44	S	26.5 ± 0.4
β-pinene	273.1	0.4	96.7 ± 3.3	80.28	S	25.9 ± 0.9
terpinen-4-ol	64.4	0.1	76.7 ± 3.3	33.61	S	29.8 ± 0.8
α-terpenene	265.8	0.4	96.7 ± 3.3	80.28	S	24.2 ± 0.8
γ-terpinene	218.3	0.3	93.3 ± 3.3	71.11	S	25.8 ± 1.4
α-terpineol	42.5	0.1	53.3 ± 13.3	4.44	S	33.7 ± 1.3
thymol	15.7	0.04	40.0 ± 5.8	0.00		30.1 ± 1.5
verbenone	101.3	0.2	63.3 ± 3.3	13.61	S	32.5 ± 1.3

[a]LD_{20} value of each compound (μg/insect)

[b]mixing ratio of each compound in the binary mixture of eugenol (w:w)

[c]individual mortality of camphor was 16.7% ± 6.7

[d]the amount of camphene and caryophyllene oxide was set to 500 μg

Supplementary Video

Toxic response of thymol and *trans*-anethole. At the applications of LD_{50} dose, while the larvae treated with thymol showed slowed movements leading to flaccid paralysis, *trans*-anethole administration produced unique tremors at the both ends, followed by paralysis (5 min after topical application).
